# Telephone-Based Structured Communication Simulation Program for the Follow-Up of COVID-19 Cases and Contacts in Primary Care

**DOI:** 10.3390/ijerph19073915

**Published:** 2022-03-25

**Authors:** María Gracia Adánez-Martínez, Ismael Jiménez-Ruiz, César Carrillo-García, José Luis Díaz-Agea, Antonio Jesús Ramos-Morcillo, Alonso Molina-Rodríguez, María Ruzafa-Martínez, César Leal-Costa

**Affiliations:** 1 Faculty of Medicine, University of Murcia, El Palmar, 30120 Murcia, Spain; g.adanez@um.es; 2 Faculty of Nursing, University of Murcia, El Palmar, 30120 Murcia, Spain; cesarcarrillo@um.es (C.C.-G.); ajramos@um.es (A.J.R.-M.); alonso.molina@um.es (A.M.-R.); maruzafa@um.es (M.R.-M.); 3 Professional Development Unit, General Directorate of Human Resources, Murcian Health Service, 30003 Murcia, Spain; 4 Faculty of Nursing, Catholic University of Murcia, Guadalupe, 30107 Murcia, Spain; jluis@ucam.edu

**Keywords:** COVID-19, communication training, telephone communication, clinical simulation, primary care

## Abstract

(1) The COVID-19 pandemic has had many consequences on health systems worldwide. In the Spanish health system, telephone-based consultations were coupled to in-person consultations. This type of consultation was mainly a challenge for the primary care teams, who had to assume the greatest load of care provision. The objective of the present study was to discover the satisfaction and perception of health professionals related to a training program on efficient communication based on high-fidelity simulation. (2) Methods: A cross-sectional descriptive study based on a convergent and parallel mixed method. The satisfaction and perception of 275 health professionals associated with COVID-19 training based on the structured communication model CERCAR© was analyzed. (3) Results: The assessment of the satisfaction with the training and methodology was high. With respect to the transfer of information, the participants gave a high score to the categories of consolidation of learning, applicability to their work, and benefits for the institution. The qualitative results supported these findings. (4) Conclusions: The training program and its virtual modality were well received, and had a high degree of transference. The application of active, online learning methodologies is a relevant format for continuous education.

## 1. Introduction

Since the World Health Organization (WHO) declared the pandemic due to SARS-CoV-2, which causes the COVID-19 disease [1], the number of people infected has exponentially increased around the world, with millions of infections and many deaths every day, which caused the collapse of health services in countries where healthcare is a common good [2,3]. Until today (11 February 2022), there have been 404,910,528 million people infected, including 5,783,776 deaths [4].

In Spain, the situation has not been any different, and since the first case was detected at the end of January 2020, the number of diagnosed (10,555,196) and dead (95,606) individuals has increased [4,5]. The health crisis caused by the pandemic has had important consequences on health systems worldwide, including Spain, with unprecedented levels of overload [6,7]. Spain was one of the most affected countries, especially during the first wave and the current sixth wave, propitiated by the virus variant Omicron (variant B.1.1.529) [8]. In the sixth wave, the symptoms of vaccinated individuals were mostly mild, characterized by nasal secretions, headache, and tiredness, as the most common Omicron symptoms [9].

Since the start of the pandemic, primary care (PC) has had to restructure its functions in record time, changing the organization of the health centers, and prioritizing the detection of infected individuals and their close contacts, without disregarding the safety of the patients and the workers in these centers [10]. Presently, a great number of the notified COVID-19 cases are mild. Therefore, the diagnosis and follow-up of the mild cases, as well as their epidemiological monitoring, must be conducted in PC, in a coordinated manner with public health and preventive medicine services [11]. The PC professionals have made great efforts to monitor the community cases, as well as those from residential homes [7].

PC, as the entryway to the health system, has had to increase their efforts, many times without an increase in resources, by adding telephone consultations to in-person ones, a modality that was hardly utilized until now and was challenging for PC teams as they had to deal with a type of consultation for which they had not been strongly trained in [12,13,14]. The telephone-based monitoring of the patients created a great uncertainty in the health professionals, given their lack of experience and the possibility of making mistakes. Furthermore, PC had to deal with a high number of consultations implemented to detect and monitor infected individuals and their close contacts, including the hiring of health personnel, who at times did not have the experience necessary. Telematic consultations had been, until the pandemic, only utilized in exceptional, non-planned, and non-routine cases. Other studies have described the need for prior training before a high-quality telephone interview can be conducted [15,16,17]. Thus, a necessity appeared for training on telephone-based communication applied to COVID-19 that could provide standardized training, safety, homogeneity in the actions, and an efficient communication foundation, with special attention to aspects such as closeness, empathy, respect, and personalized attention, which are associated with a higher degree of therapeutic compliance [18,19].

Many years ago, the WHO launched nine recommendations for the safety of the patient, among which we find communication training of health professionals [20]. Structured communication protocols have been widely proven to increase the safety of the patient, preventing the loss of important informational contents, as systematically recommended by highly experienced scientific societies, such as the European Resuscitation Council (ERC), which recommends the use of the SBAR (situation, background, assessment, recommendations) protocol [21]. Numerous studies have also described the beneficial effects of structured communication protocols on the safety of the patient [22,23,24].

In the Region of Murcia (Spain), a structured communication tool was designed to facilitate the telephone-based monitoring and follow-up of patients diagnosed with COVID-19 with mild–moderate symptoms and their close contacts, by PC teams. The tool was named CERCAR© COVID-19 (the Spanish acronym means “surround or circumvent something so that it is separated from others” [25], which is applicable to what is intended with the protocol, that is, to fence in the propagation of the virus). It was created with the consensus of five experts in health communication and the monitoring and follow-up of COVID-19 patients [26]. The structured communication protocols, for the first telephone-based interview, as well as for the follow-up, can be viewed in Table S1.

As of today, there is no doubt that clinical simulation is an essential component in health sciences [27], and as described in recent articles, it has been shown to be an effective learning methodology during the pandemic [28,29]. Likewise, clinical simulation has shown to be an effective methodology for the learning of structured communication protocols [30,31]. In response to the training needs detected in PC, due to the pandemic, we suggest an innovative proposal of simulation that recreates high-fidelity scenarios through simulated online telephone calls, for the monitoring and follow-up of patients diagnosed with COVID-19 with mild–moderate symptoms, as well as their close contacts.

This study was designed to focus on adapting the training based on clinical simulation to the COVID-19 situation of PC health professionals. The objective of the present study was to describe a training program based on the structured communication protocol CERCAR© COVID-19, and to determine the satisfaction of the PC health professionals, as well as their perceptions, after they were trained with this innovative online training methodology.

## 2. Methods

### 2.1. Design

A cross-sectional descriptive study was conducted. A convergent, parallel mixed method (with quantitative and qualitative data collection in the same phase) [32] was used to analyze the satisfaction and perceptions of participants in the training program based on the structured communication model CERCAR© COVID-19 through clinical simulation. Qualitative data were collected to supplement and provide further insights into the quantitative data.

### 2.2. Setting and Sample

The sample was composed by health professionals (physicians and nurses) from PC in the Region of Murcia, Spain. All of them participated in training sessions based on high-fidelity clinical simulation.

The sampling method was convenience sampling. The inclusion criteria for being part of the study were: (1) PC health professionals who conducted or would conduct monitoring and follow-up of COVID-19 patients with mild–moderate symptoms and their close contacts, (2) participation in the training program based on the CERCAR© COVID-19 structured communication program, and (3) signing the informed consent form. Participants who abandoned the training program within the data-gathering period were excluded.

The program was offered to the participants as training that was part of the strategic plan on COVID-19 of the Murcian Health Services, Spain. The participants enrolled in the course through an online platform. A total of 19 sessions were conducted, with a maximum of 20 participants per course, between October 2020 and November 2021.

### 2.3. Training Program

A training program with active learning methodologies was designed. The training was planned in two differentiated online modalities.

#### 2.3.1. Asynchronous General Theoretical Training

This was conducted in the training platform from the Murcian Health Services. Specific materials were created for clinical training on COVID-19, in the form of voiced-over videos (Table 1), which included one on efficient communication with the CERCAR© COVID-19 structured communication protocol. All the participants had to comply with the asynchronous online portion and pass an exam before taking part in the practical training.

#### 2.3.2. Synchronous Practical Training

This was conducted with 1 session on Zoom, which lasted 4 h and was eminently practical.

Different active learning methodologies, group dynamics, reflective analyses of problem videos, and high-fidelity clinical simulations were combined. There was a constant interaction between students and professors. The previous theoretical contents were successfully obtained before, so that reflection-based tasks could be conducted through the analysis of problem videos and simulated telephone interviews during the practical session.

##### Simulation Design Process

The simulation program was designed following the International Nursing Association of Clinical and Simulation Learning (INACSL) standards [33].

**Figure 1:**
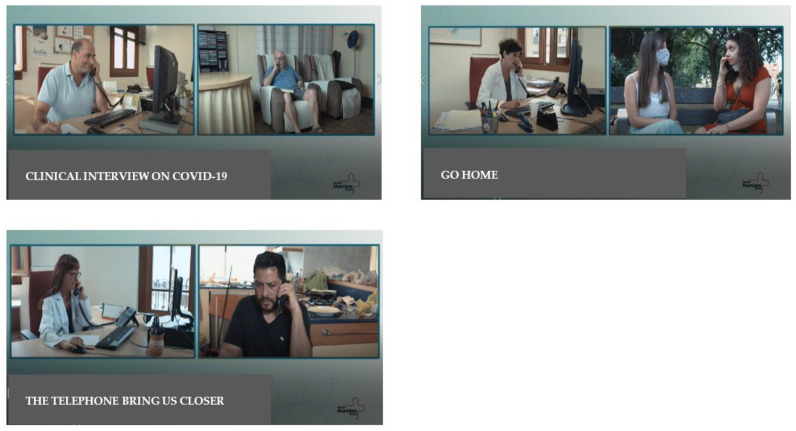
Teaching/problem videos of clinical cases with COVID-19 patients.

The videos were designed by a panel of 3 experts in active learning methodologies, health communication, and the monitoring and follow-up of COVID-19 patients. Situations were selected which tended to show frequent dilemmas in the making of clinical decisions. Different situations were scripted in which the PC health professionals made telephone calls for the monitoring and tracking of COVID-19 patients. Three videos were created which showed different clinical situations. These were recorded with the participation of professional actors and actresses and volunteer physicians and nurses (Table 2 and Figure 1). The recording of the videos was conducted with COVID funds from the professional development unit from the Murcian Health Services (Spain). Two types of videos were created: (1) teaching videos which illustrated a telephone interview conducted correctly through the use of the structured communication protocol CERCAR © COVID-19; and (2) problem videos showing unfinished situations, in which the students had to identify the problem, reflect, and seek solutions.

Twenty clinical scenarios were designed in the telephone-based interviews with diverse situations which presented clinical and social problems associated with the monitoring and follow-up of COVID-19 patients and their close contacts (Table 3). All the scenarios were designed following the most accepted international recommendations for the design of scenarios [33] and, more specifically, the proposals by Moral and Maestre [34], with the following stages: (1) a study of the training needs; (2) a definition of the learning objectives; (3) an agenda and plan of the scenario; and (4) a selection of the debriefing style. The standardized patients were selected and trained for playing the roles to guarantee a standardized process and a high degree of fidelity of the scenarios.

##### Prebriefing

The INACSL indications [35] were followed. The first half hour of the session was utilized to establish a psychologically safe environment. For this, various group dynamics were established, based on the practices proposed by Rudolph, Raemer, and Simon [36], and the good practices standards from the INACSL [33] were used to establish a psychologically safe environment. The dynamics utilized were:

1. Detailed explanation of the development of the session.
2. Clarification of the expectations and the answering of questions posed with respect to the development of the online simulation session.
3. Explanation of the logistic details of the platform and the tools utilized.
4. Explanation that a mistake is an opportunity to learn (the mistakes do not have risk or consequences).
5. Establishment of a “fictional contract” with the participants.
6. Agreement of confidentiality and commitment of respect to the other participants.

These dynamics were conducted to comply with the three attributes of the psychological learning environment [37]: (1) the ability to make mistakes without consequences; (2) the qualities of the facilitator; and (3) fundamental activities, such as orientation, preparation, objectives, and expectations.

##### Teaching Problem Videos

Once a psychologically safe environment was achieved, the analysis of the videos began. In first place, the participants watched the “typical” interview learning video which illustrated the application of the CERCAR© COVID-19 structured communication protocol. Afterwards, they watched the problem videos, where the participants had to identify the problem, reflect, and look for solutions, utilizing the structured debriefing method [38]. For this, the student had to assume the role of the health professionals in the video.

##### Simulated Telephone Interviews

Once the training with the teaching problem videos had ended, simulated telephone-based interviews were conducted. This phase was divided into the following stages. 1) Briefing. Before each simulation scenario, the information about the scenario proposed was presented to the participants. 2) Simulated telephone-based interview. The facilitators turned the camera off and adopted the role of the simulated patient, creating the scenario of a telephone call, in which the student played the role of a health professional, having to make diverse clinical and social decisions, and manage resources. The observing students created a checklist about the positive aspects and those that could be improved to favor the analysis and reflection of the case. 3. Debriefing. When the simulated telephone-based interview ended, a structured debriefing took place [38], which included a discussion on how to transfer the knowledge acquired to real situations. For the debriefing, the INACSL indications were followed [39].

### 2.4. Data Collection Instrument

Socio-demographic (age and marital status) and professional (professional category, resident in training, and time at position) characteristics were assessed.

To determine the satisfaction of the participants with the simulated telephone-based interviews, the Satisfaction Scale questionnaire with high-fidelity clinical simulation [40] was utilized. This questionnaire is composed by 33 items, and uses a Likert-type scale with 5 response options, from “strongly disagree” to “strongly agree”, with the response to item 25 inverted, as it was written in the negative sense. After its completion, a good internal consistency was obtained (Cronbach’s alpha = 0.977), i.e., a similar value to that obtained in the original scale (Cronbach’s alpha = 0.920). The values of the item–total correlation ranged between 0.54 and 0.85, i.e., a value similar to that obtained in the original scale (0.52 and 0.73).

Then, 40 days after the practical training, six questions were sent to the participants to assess the transfer of the training to their position at work. For this, the following aspects were measured: the consolidation of the learning (3 questions), the applicability to the position at work (4 questions), and benefits of the organization (1 question). The questions were answered with a 5-option Likert-type scale, ranging from “strongly disagree” to “strongly agree”.

Lastly, the perceptions of the participants related with the training program were explored through an open-ended question about their opinions on their experience with the online simulation: “In a few lines, write your sincere opinion about the simulation-based training, positive aspects, or improvements you believe should be made”.

### 2.5. Statistical Analysis

The data were analyzed with the IBM SPSS Statistics version 22.0 for Windows (IBM Corp., Armonk, NY, USA) The descriptive statistical values were calculated (frequency and percentages for the categorical values, and mean and standard deviation for the quantitative values). To analyze the mean differences between the socio-demographic and professionals variables used with respect to the professional category variable, Student’s t-tests and chi-square tests were employed depending the nature of the variable compared. Before the Student’s t-tests were performed, the assumptions of normality of the data were verified using the Kolmogorov–Smirnov test.

The qualitative data obtained from the open-ended question were analyzed independently by two different members of the research team (with extensive experience in qualitative research, i.e., a Ph.D. in Psychology and a Ph.D. in Medicine), using the open-coding strategy [41]. A consensus was established of the final categories through a thematic analysis. The qualitative results were integrated within the quantitative results to provide them with context. The chosen codification system for the informants was (D. #) for physicians and (N. #) for nurses.

### 2.6. Ethical Considerations

The approval by the Ethics Committee from the University Clinical Hospital Virgen de la Arrixaca, Spain was obtained (Number NE-2021-3-HCUVA). The objectives of the study were described previously, and all the participants provided their written informed consent to participate in the study. The study was conducted following the standards and recommendations from the International Declaration of Helsinki [42].

## 3. Results

A total of 275 health professionals (physicians and nurses) from PC participated in the study (72.4% response rate). The profile of the health professionals was women (*n* = 205; 74.5%), aged between 22 and 70 years (M = 32.2; SD = 9.9), with a marital status of single *n* = 202; 73.5%). As for their professional characteristics, many were physicians (*n*= 156; 56.7%), with a mean of 62.5 months of professional experience (SD = 241.1), and most were residents training in medicine or nursing (*n* = 214; 77.8%) (Table 4).

The descriptive analysis of the items from the satisfaction with training based on a clinical simulation questionnaire is shown in Table 5. As observed, all the items obtained a mean score higher than 4.5 out of 5, except for item 5 (“the degree of difficulty of the cases was appropriate for my amount of knowledge”) and item 25 (“I lost my temper in some of the cases”). In addition, statistically significant differences were not found between the physician and nurse scores, although for the latter, they were slightly higher in almost all the items.

**Figure 2:**
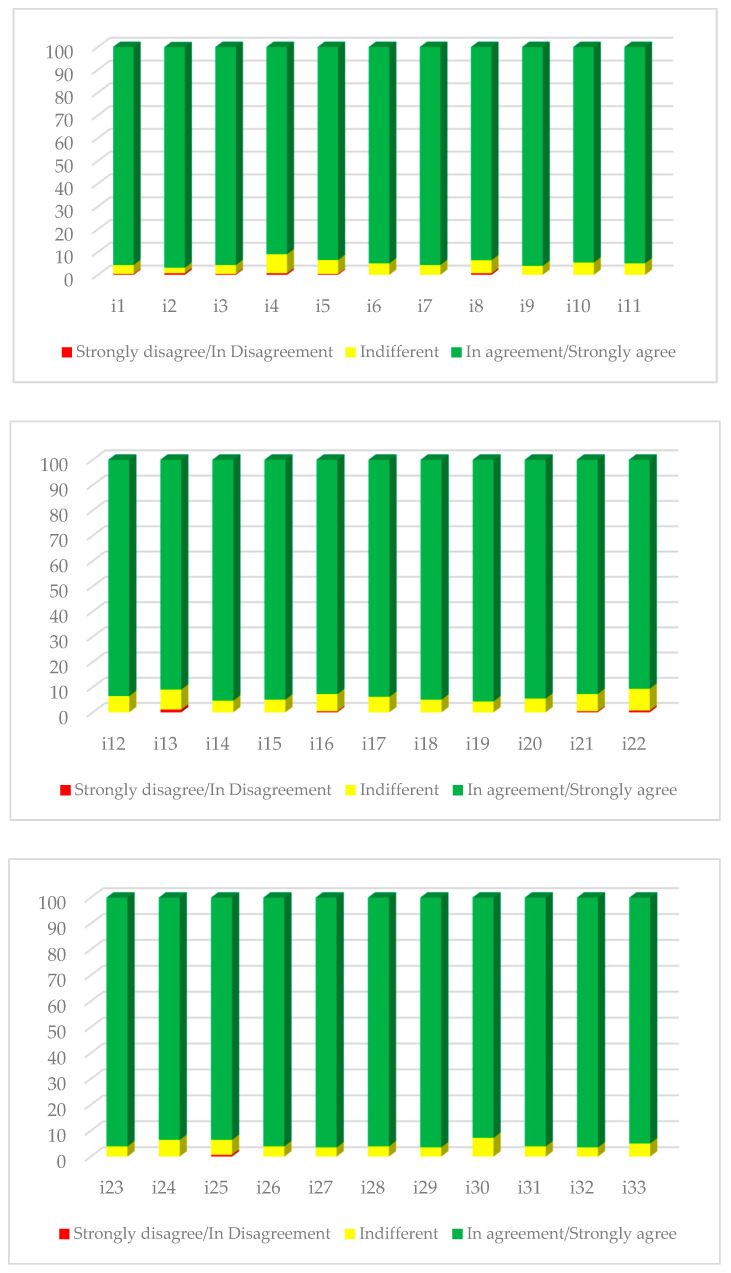
Response percentages for items of the clinical simulation satisfaction questionnaire.

To facilitate the understanding of the results, Figure 2 shows the percentages of the responses, with response scales grouped in the following manner: strongly disagree, in disagreement, indifferent, in agreement, and strongly agree. In all the items, the scores obtained in the scale “strongly agree/completely agree” were higher than 90%. In 13 items from the questionnaire, percentages >95% were obtained (items 1, 2, 3, 7, 9, 14, 19, 23, 26, 27, 29, 31, and 32). The lowest percentages (<91%) were obtained in items 4, 13, and 22.

Forty days after the training, the participants were asked about the transfer of the training to their work, with 145 participants providing an answer. As observed in Table 6, the participants provided a high score to the questions related with the consolidation of the learning (between 4.3 and 4.44 out of 5), applicability to their work position (between 4.25 and 4.38 out of 5), and benefit to the organization (4.48 out of 5). If we focus on the scores according to professional category, the physicians provided higher scores as compared to the nurses. Statistically significant differences were not found in most of the items, except for items 5 (“the training has helped you in the making of decisions related to COVID-19 in your position at work”) and 6 (“I’ve had the opportunity at work to participate in the telephone-based monitoring of COVID-19 cases”) from the dimension applicability to the position at work.

Lastly, the open-ended question about the training based on clinical simulation allowed all the participants to express their opinions in the shape of short comments or phrases. Five categories were identified, which were organized according to frequency of mention: (1) satisfaction with the experience based on clinical simulation; (2) transfer to clinical practice and the use of the CERCAR© COVID-19 structured communication protocol; (3) learning in a psychologically safe environment; (4) fidelity of the simulation; and (5) benefits of reflection and debriefing. Table 7 shows extracts of significant quotes as examples of all the categories identified.

## 4. Discussion

The present study presents a training program based on online clinical simulation to provide an answer to the impossibility of providing in-person training to PC health professionals, due to the restrictive measures imposed by the COVID-19 pandemic. The program provided a high degree of satisfaction and consolidation of learning, as evaluated by the health professionals.

Our objective was to conduct simulated telephone-based calls for the monitoring and follow-up of patients diagnosed with COVID-19 with mild or moderate symptoms, as well as of their close contacts, which had to comply with the requisites proposed by the INACSL [33] during their execution. Given that the training was online, some standards had to be modified, such as that the scenario did not take place in a simulation room adapted to simulate a primary care consultation; thus, this experience was based on simulated online phone calls instead.

This experience was coherent and adapted to the reality of clinical practice in PC services, where little training is provided for this type of telephone-based consultation, although it was highly utilized during the pandemic [12,13,14,15,16,17].

The high degree of satisfaction and the positive comments from the participants about the training program were strongly associated, underlining a high degree of satisfaction. These results were congruent with different studies that utilized in-person [40,43,44,45] and online [46,47,48] clinical simulation methodologies. The results from the present study showed that the items that were best scored by the participants were related with a psychologically safe environment (item 6—“I felt comfortable and respected during the sessions”), the fidelity of the clinical scenarios (item 3—“Cases recreated real situations), and the usefulness of reflection and debriefing (items 27—“The teacher provided constructive feedback after each session”, 28—“Debriefing has helped me reflect on the case”, and 29—“Debriefing at the end of the session has helped me correct mistakes”), where the participants provided mean scores of >4.70 out of 5. These results were congruent with those obtained in the qualitative analysis, and this was possible because the training program followed international recommendations for the design of activities based on clinical simulation [33], the use of a psychologically safe environment [36,37], and a structured debriefing [38,39]. The analysis of the satisfaction with the training according to the variable professional category highlighted the lack of statistically significant differences between the scores provided by the physicians and nurses, although the scores provided by the nurses were slightly higher in almost all the items.

The main advantage of simulation-based training was its ability to link theory with practice and to learn from one’s mistakes. These aspects were underlined by the PC health professionals in the satisfaction questionnaire and verbatim (in their comments), which is in agreement with previous studies [49,50,51]. Likewise, taking part in a simulation, even a virtual one, allowed them to learn as a team about experiences that must be resolved as a team, as in a real work environment [52].

The training program was based on the CERCAR© COVID-19 structured communication protocol [26], for the telephone-based interview of patients infected with COVID-19 with mild–moderate symptoms and their close contacts. This communication protocol facilitated the interview by the PC health professionals, as it helped them to avoid forgetting to ask relevant information, as shown in the verbatim comments from the participants. These data are similar to results from other studies, which provided evidence of the beneficial use of structured communication protocols on the safety of the patient [22,23,24]. The participants stated that the simulated telephone calls improved their communication skills, and that the CERCAR© COVID-19 structured communication protocol helped them to conduct the interviews through the use of efficient communication.

As for the fidelity of simulated scenarios, the PC health professionals indicated that the simulation experience was realistic, due to the fact that the simulated scenarios were contextualized to the real-world situation, and promoted the transfer of knowledge and skills to clinical practice. The achievement of a high fidelity in the simulated scenarios was due to use of standards used in the design of the training based on simulation and the design of scenarios [33]. To achieve this objective, the facilitators turned their cameras off so that the health professional giving the interview could focus on the audio, as in a real telephone call, and the standardized patient was trained in each of the clinical scenarios. This aspect is very important, as greater fidelity used in the simulation-based activities led to a greater impact on the learning results of the activity [53]. Another aspect that was evaluated by the participants was the transfer of the simulation-based training to their position at work. All the professionals gave high scores to the questions about consolidation of learning (between 4.26 and 4.44 over 5), applicability to the position at work (between 4.25 and 4.38 over 5), and benefit for the organization (4.48 over 5). This is essential, as the training had a great impact on the clinical practice of the professionals, who stated that their theoretical-practical knowledge and communications skills had increased, and they had applied everything they had learned to their work (including the CERCAR© COVID-19 structured communication protocol). These results are congruent with other studies, where a transfer of the simulation-based training to clinical practice was evidenced [54,55].

The participants expressed having received constructive feedback after each session, and the debriefing phase helped them to reflect on the cases and correct their mistakes. These results were consistent with most of the evidence found [38,56,57].

Lastly, telephone-based communication presents a range of ethical challenges. Some of the ethical issues to consider in telehealth, and more specifically in telephone-based communication, are privacy, security, confidentiality, liability, and so forth [58,59]. The positive ethical implications of using a telephone approach included providing respondents privacy, respondents choosing convenient interview times, and affording health providers more privacy than institutional in-person interviews [59]. These positive aspects coincide with the results of our study, where the time limits on call duration were flexible to allow for active listening and empathetic inquiry.

### Limitations

This study is not without limitations. A methodological limitation was the convenience sampling, although the sample size was high and representative, as a great participation was observed from PC health professionals in the simulation-based training. Another limitation was the technical problems during the videoconferences. However, these problems are common to real telephone calls, given the lack of coverage, so that on some occasions, this made the scenarios more realistic. Lastly, most of the sample was composed by nurses and resident physicians, who were still in training. However, training the residents encouraged more PC physicians and nurses to use the CERCAR© COVID-19 structured communication protocol as a way of monitoring infected individuals and their close contacts, after observing how it worked and how it was used by the residents who had taken the course.

## 5. Conclusions

This online simulation-based training activity, which recreated high-fidelity scenarios through simulated telephone calls, provides a response to the education needs of PC health professionals provoked by the COVID-19 pandemic. In this sense, the study shows that this continuous training, in its virtual modality through high-fidelity simulation, is plausible and welcomed by the health professionals, as it has a high degree of consolidation of learning, among other aspects.

The participants satisfactorily evaluated the training experience, and it had a high rate of transfer to clinical practice. The high-fidelity online simulation is a useful option, not only in the current situation of COVID-19, but also in other clinical contexts, in which in-person training activities are not possible, or to favor the accessibility and aspects such as social or family conciliation.

To conclude, given the advantages of the CERCAR© COVID-19 structured communication protocol and the training program, it will be presented to other health systems in other regions of Spain for its possible implementation.

## Figures and Tables

**Table 1:** Voiced-over videos.

Module 1	Basic aspects of COVID-19
Module 2	Basic aspects of monitoring of COVID-19 patients and their contacts
Module 3	Steps to take for identifying cases and investigate their contacts
Module 4	Ethical/legal aspects in the management of COVID-19 patients
Module 5	Skills needed to achieve effective communication

**Table 2:** Teaching/problem videos of clinical cases of monitoring and follow-up of COVID-19 patients.

Video Title	Summary	Objectives
Clinical interview on COVID-19	Clinical telephone-based interview of COVID-19 patient.	- Apply the CERCAR protocol. - Learn effective communication tools.
The telephone bring us closer	Clinical interview of a COVID-19 patient with social problems.	- Apply the CERCAR protocol. - Resolution of situations with a social problem. - Use of community resources.
Go home	Clinical interview of a patient who is possibly not complying with confinement.	- Apply the CERCAR protocol. - Resolution of problems with difficult patients. - Efficient communication skills.

**Table 3:** Clinical cases.

Scenario	Learning Objectives
You call Maria, her six-year old son Abel is waiting for PCR results due to a close contact at school, his PCR is positive	- Apply the CERCAR© protocol - Making of decisions, isolation
You call Mario, 43 years old. You inform him that his PCR test is positive.	- Apply the CERCAR© protocol - Making of decisions. - Confidentiality.
You call Javier, 18 years old. Works in a private residential home, had a PCR test 24 h ago and is positive.	- Apply the CERCAR© protocol - Making of decisions - Isolation - COVID protocol for health workers
You call Yesica, 20 years old. PCR positive. The test was done 24 h ago.	- Apply the CERCAR© protocol - Making of decisions - Confidentiality
You call Manuel, 36 years old, positive PCR. Test done 48 h ago. Negationist.	- Apply the CERCAR© protocol - Making of decisions - Negationism
You call Habib. Positive PCR. Does not understand the language. You hear lots of coughing. Important communication barrier. A 10 year-old girl gets the phone.	- Apply the CERCAR© protocol - Making of decisions - Language barrier
You call Antonia, 82 years old. Follow-up call due to positive PCR test, it’s the third day you call.	- CERCAR© protocol - Severe worsening of general health - Making of decisions
You call Ruben for a follow-up. 20 years old. Positive PCR 10 days ago, asymptomatic on yesterday’s call.	- CERCAR© protocol - Re-enforce isolation in the last few days.
You call Tomas, 50 years old, for a follow-up. Positive PCR 4 days ago.	- CERCAR© protocol - Mild worsening of general health - Making of decisions and COVID-19 protocol
You call Eugenia, 50 years old. Very symptomatic. Negative PCR.	- CERCAR© protocol - High clinical suspicion with negative PCR. - High-anxiety situation
You call Pedro, 18 years old, because he was a close contact. Positive PCR yesterday.	- CERCAR© protocol - Breach of quarantine
You call Felipe, 56 years old. Mild symptoms. He is homeless and lives on the street.	- CERCAR© protocol - Social problem - Social health resources available
You call Julia, 22 years old. Does not answer the calls.	- CERCAR© protocol - Resources and action protocol against breach of quarantine/no answer
You call Ana, in quarantine due to close contact 10 days ago. She was just deemed positive.	- CERCAR© protocol - Making of decisions - Efficient communication skills
You call Carmen to inform her she is PCR positive. She just came back from her father’s funeral, who died due to COVID-19.	- CERCAR© protocol - Making of decisions - Efficient communication skills
You call Ruben, nursing student. He is positive. He already knows it because his girlfriend, a nurse, has just informed him.	- CERCAR© protocol - Ethical-legal problems, confidentiality - Efficient communication skills
You call Abel, 85 years old. His daughter answers the phone. She informs you that he has just passed away.	- CERCAR© protocol - Efficient communication skills - Action protocol of patients who have passed away
You call Mario, 43 years old, he was just discharged from the hospital. He was admited due to pneumonia due to COVID-19.	- CERCAR© protocol - Efficient communication skills - Monitoring and follow-up protocol
You call Javier, 23 years old. He has received two doses of the vaccine. He is positive due to close contact.	- CERCAR© protocol - Efficient communication skills - Follow-up protocol
You call Monica, caregiver to the elderly in a residential home, she is a close contact. She resists vaccination.	- CERCAR© protocol - Efficient communication skills - Monitoring and follow-up protocol

**Table 4:** Participants’ characteristics.

	Total *n* = 275	Groups		*p*
		Physician *n* = 156	Nurse *n* = 119	
Age M (SD)	31.2 (9.9)	29.6 (8.2)	33.3 (11.5)	0.003 ^a^
Gender *n* (%)				
Women	205 (74.5)	110 (70.5)	95 (79.8)	0.08 ^b^
Men	70 (25.5)	46 (29.5)	24 (20.2)	
Marital status *n* (%)				
Single	202 (73.5)	125 (80.1)	77 (64.7)	0.004 ^b^
Married	69 (25.1)	28 (17.9)	41 (34.5)	
Divorced	3 (1.1)	3 (2.0)	0 (0)	
Widower	1 (0.4)	0 (0)	1 (0.8)	
Resident in training *n* (%)				
Yes	214 (77.8)	141 (90.4)	73 (61.3)	0.000 ^b^
No	61 (22.2)	15 (9.6)	46 (38.7)	
Professional experience months M (SD)	62.5 (241.1)	26.1 (73.1)	110.2 (352.1)	0.012 ^a^

Note: a = *t* for continuous quantitative values, and b = *X^2^* for qualitative variables; M= mean; SD= standard deviation.

**Table 5:** Descriptive analysis for items of the clinical simulation satisfaction questionnaire.

Items M (SD)	Total *n* = 275	Groups		*p*
		Physician *n* = 156	Nurse *n* = 119	
1. Facilities and equipment were real	4.64 (0.58)	4.62 (0.62)	4.68 (0.52)	0.35
2. Objectives were clear in the cases	4.73 (0.56)	4.72 (0.56)	4.75 (0.56)	0.73
3. Cases recreated real situations	4.72 (0.55)	4.71 (0.59)	4.74 (0.49)	0.68
4. Timing for each simulation case was adequate	4.53 (0.68)	4.50 (0.70)	4.56 (0.65)	0.45
5. The degree of difficulty of the cases was appropriate for my amount of knowledge	4.45 (0.63)	4.46 (0.60)	4.45 (0.66)	0.90
6. I felt comfortable and respected during the sessions	4.77 (0.53)	4.79 (0.51)	4.74 (0.56)	0.45
7. Clinical simulation is useful for assessing a patient’s clinical simulation	4.68 (0.55)	4.66 (0.57)	4.71 (0.53)	0.50
8. Simulation practices help you learn to avoid mistakes	4.53 (0.64)	4.53 (0.64)	4.53 (0.65)	0.97
9. Simulation has helped me to set priorities for action	4.67 (0.55)	4.65 (0.57)	4.71 (0.53)	0.38
10. Simulation has improved my ability to provide care to my patient	4.67 (0.58)	4.67 (0.57)	4.66 (0.58)	0.97
11. Simulation has made me think about my next clinical practice	4.69 (0.56)	4.69 (0.56)	4.70 (0.56)	0.94
12. Simulation improves communication and teamwork	4.63 (0.60)	4.62 (0.61)	4.65 (0.59)	0.73
13. Simulation has made me more aware/worried about clinical practice	4.52 (0.69)	4.53 (0.70)	4.51 (0.67)	0.82
14. Simulation is beneficial for relating theory to practice	4.71 (0.55)	4.71 (0.56)	4.70 (0.54)	0.83
15. Simulation allows us to plan the patient’s care effectively	4.59 (0.59)	4.57 (0.61)	4.62 (0.55)	0.47
16. I have improved my technical skills	4.58 (0.64)	4.55 (0.65)	4.62 (0.61)	0.36
17. I have reinforced my critical thinking and decision-making skills	4.63 (0.60)	4.60 (0.61)	4.66 (0.58)	0.40
18. Simulation helped me assess a patient’s condition	4.56 (0.59)	4.52 (0.59)	4.61 (0.58)	0.19
19. This experience has helped me prioritize care	4.61 (0.57)	4.57 (0.59)	4.66 (0.54)	0.22
20. Simulation promotes self-confidence	4.64 (0.58)	4.61 (0.61)	4.67 (0.55)	0.37
21. I have improved communication with the team	4.56 (0.64)	4.57 (0.62)	4.55 (0.66)	0.84
22. I have improved communication with the family	4.52 (0.69)	4.50 (0.70)	4.55 (0.66)	0.58
23. I have improved communication with the patient	4.72 (0.53)	4.71 (0.54)	4.73 (0.52)	0.76
24. This type of practice has increased my assertiveness	4.63 (0.60)	4.60 (0.64)	4.68 (0.55)	0.25
25. I lost my temper in some of the cases	4.35 (0.62)	4.38 (0.58)	4.31 (0.67)	0.38
26. Interaction with simulation has improved my clinical competence	4.61 (0.56)	4.61 (0.56)	4.61 (0.57)	0.95
27. The teacher provided constructive feedback after each session	4.77 (0.50)	4.76 (0.51)	4.78 (0.49)	0.68
28. Debriefing has helped me reflect on the cases	4.75 (0.52)	4.78 (0.50)	4.71 (0.54)	0.33
29. Debriefing at the end of the session has helped me correct mistakes	4.76 (0.51)	4.77 (0.51)	4.74 (0.51)	0.63
30. I knew the cases’ theoretical side	4.45 (0.63)	4.47 (0.63)	4.43 (0.63)	0.61
31. I have learned from the mistakes I made during the simulation	4.71 (0.53)	4.70 (0.54)	4.73 (0.53)	0.62
32. Practical utility	4.73 (0.52)	4.71 (0.53)	4.76 (0.50)	0.48
33. Overall satisfaction with the sessions	4.65 (0.57)	4.63 (0.59)	4.67 (0.55)	0.59

**Table 6:** Descriptive statistics of the questions about consolidation of learning, the applicability to the position at work, and the need for additional training.

Items M (SD)	Total *n* = 145	Groups		*p*
		Physician *n* = 92	Nurse *n* = 55	
Consolidation of the learning				
1. The training has improved your theoretical-practical knowledge in relation to COVID-19.	4.44 (0.66)	4.47 0.67	4.38 (0.65)	0.45
2. It has implied an improvement in your technical competences.	4.36 (0.66)	4.40 (0.63)	4.29 (0.71)	0.33
3. The training has implied an improvement in your communication skills and the achievement of efficient communication.	4.37 (0.65)	4.41 (0.67)	4.29 (0.63)	0.27
Applicability to the position at work				
4. You have had the opportunity to apply the knowlede acquired related with COVID-19 at work.	4.33 (0.72)	4.41 (0.71)	4.20 (0.73)	0.08
5. The training has helped you in the making of decisions related to COVID-19 in your position at work	4.32 (0.71)	4.41 (0.67)	4.16 (0.76)	0.04
6. I’ve had the opportunity at work to participate in the telephone-based monitoring of COVID-19 cases	4.38 (0.73)	4.50 (0.62)	4.17 (0.84)	0.01
7. You have used the CERCAR© COVID-19 protocol in the monitoring of cases and close contacts.	4.25 (0.65)	4.33 (0.65)	4.13 (0.64)	0.07
Benefit for the organization				
8. Do you believe the training has been beneficial to the Organization?	4.48 (0.61)	4.51 (0.60)	4.42 (0.63)	0.38

**Table 7:** Verbatim text of the categories identified after the thematic analysis of the participant’s comments.

Category	Verbatim
Satisfaction with the experience based on clinical simulation	“I loved the course, it was very useful. The professors promoted interaction, dynamics, and trust!”(N40) “Thank you for this Learning” (N54) “It was a very complete, interesting, and stimulating course” (D74) “I consider the simulation sessions to be especially useful for our training” (D104) “Very useful and pertinent course at this moment in time” (D116) “The execution of the simulation sessions was very entertaining. Mi sincere congratulations to the professors” (D128) “Four very entertaining hours, I had never taken an online course that was this interactive. Congratulations to everyone” (D147) “Very good course and good simulation” (D183) “Very happy with this online initiative, I hope it is done more” (D199)
Transfer to clinical practice and the use of the structured communication protocol CER-CAR© COVID-19	“The CERCAR protocol allowed me to not forget any key questions for my patients” (D40) “…it provides, in an easy manner, all the guidelines to follow to avoid our forgetting of important information, as well as confidence when making COVID-related calls” (D74) “It gives you the tools necessary for every day practice” (N57) “Thank you for organizing and teaching this course, it has greatly helped in the learning to deal with different situations that we may find daily in our consultations” (D217) “It has been a very interesting part, especially the simulation of diverse situations that we could experience in clinical practice” (D221) “Very useful and practical for my job as a physician” (D230) “...course, very necessary for physicians and nurses in primary care, who have cases of contacts or COVID+ cases, and we have to make telephone calls. I did not know the CERCAR protocol or the access to courses such as CORECAS, so I thought this course was very necessary” (N270)
Learning in a psychologically safe environment	“It is a novel methodology taught by excellent professionals, who have made learning easy“ (N131) “I am thankful for the opportunity given to us to be able to make mistakes in safe and tutored environments” (D160) “The fact that the course is interactive and participative is what makes it great” (D204) “I especially liked the practical part, as we were all able to participate, and it was therefore very dynamic”(D217) “Excellent teaching team. The dynamics they applied, as well as the videos chosen, the reflections, and the theoretical-practical part were excellent…” (D235) “The type of dynamics of the activity made it easier. It was never too long and boring, on the contrary, it was the putting into practice of what we had learned, and it allowed us to constantly participate, making these hours more entertaining. Having said that, it has exceeded my expectations” (D252) “…I want to thank the good attitude of the teaching staff, always in a good mood, without judging at any moment in time and helping us” (N270)
Fidelity of the simulation	“Excellent course; with simulations that were very similar to real life, which greatly help the putting into practice of what I had learned” (D235)
Benefits of reflection and debriefing	“…its practial nature and the debriefing helped analyze our mistakes and those of others to improve and learn…” (D155) “I liked the fact that it was participative. Debating and sharing discrepancies is always enriching. Many thanks to the professors; for making it entertaining and giving us freedom to talk” (D249)

## Data Availability

The data are available upon email request to the corresponding authors.

## Supplementary Materials

The following supporting information can be downloaded at: https://www.mdpi.com/article/10.3390/ijerph19073915/s1, Table S1: Structured communication protocol CERCAR© COVID.

## References

[1] Sohrabi C., Alsafi Z., O’Neill N., Khan M., Kerwan A., Al-Jabir A., Iosifidis C., Agha R. (2020). World Health Organization Declares Global Emergency: A Review of the 2019 Novel Coronavirus (COVID-19). Int. J. Surg..

[2] Organización Mundial de la Salud (OMS) Nuevo Coronavirus 2019. https://www.who.int/es/emergencies/diseases/novel-coronavirus-2019.

[3] Giwa A.L., Desai A., Duca A. (2020). Novel 2019 Coronavirus SARS-CoV-2 (COVID-19): An Updated Overview for Emergency Clinicians. Emerg. Med. Pract..

[4] World Health Organization (WHO) Coronavirus (COVID-19) Dashboard. https://covid19.who.int.

[5] Centro Nacional de Epidemiología Situación de COVID-19 en España. https://cnecovid.isciii.es/covid19/.

[6] Castilla J., Moreno-Iribas C., Ibero Esparza C., Martínez-Baz I., Trobajo-Sanmartín C., Ezpeleta C., Guevara M., Grupo para el Estudio de COVID-19 en Navarra (2021). Primera onda pandémica de COVID-19 en Navarra, febrero–junio 2020. Anal. Sist. Sanit. Navarra.

[7] De Nicolás Jiménez J.M., Blázquez Recio L.M., Fabregat Domínguez M.T., Palomo Cobos L. (2020). COVID-19 y esfuerzo asistencial en atención primaria. Atenc. Prim..

[8] Soriano J.B., Gerli A.G., Centanni S., Ancochea J. (2022). Forecasting COVID-19 Infection Trends and New Hospital Admissions in Spain Due to SARS-CoV-2 Variant of Concern Omicron. Arch. Bronconeumol..

[9] Iacobucci G. (2021). Covid-19: Runny Nose, Headache, and Fatigue Are Commonest Symptoms of Omicron, Early Data Show. BMJ.

[10] Gobierno de España, Ministerio de Sanidad (2020). Manejo en Atención Primaria y Domiciliaria del COVID-19.

[11] Barrio Cortes J., Mir Sánchez C., Regato Pajares P. (2021). Atención primaria en el domicilio en el marco de la pandemia COVID-19. Atenc. Prim..

[12] Mehrotra A., Ray K., Brockmeyer D.M., Barnett M.L., Bender J.A. (2020). Rapidly Converting to “Virtual Practices”: Outpatient Care in the Era of COVID-19. NEJM Catal. Innov. Care Deliv..

[13] Ludwig C., Stoevesandt D., Ludwig C., Fritsche V. (2021). Telephone-Based Communication Training in the Era of COVID-19. GMS J. Med. Educ..

[14] Sindhu K.K. (2021). The Phone: Communication in the Age of COVID-19. Patient Educ. Couns..

[15] Sales M.B., Mira M.J.M. (2020). Consulta Telefónica. Rev. Med. Fam. Aten. Primaria.

[16] Khan M.N.B. (2013). Telephone Consultations in Primary Care, How to Improve Their Safety, Effectiveness and Quality. BMJ Qual. Improv. Rep..

[17] Flint L., Kotwal A. (2020). The New Normal: Key Considerations for Effective Serious Illness Communication Over Video or Telephone During the Coronavirus Disease 2019 (COVID-19) Pandemic. Ann. Intern. Med..

[18] Pinto R.Z., Ferreira M.L., Oliveira V.C., Franco M.R., Adams R., Maher C.G., Ferreira P.H. (2012). Patient-Centred Communication Is Associated with Positive Therapeutic Alliance: A Systematic Review. J. Physiother..

[19] Zolnierek K.B.H., Dimatteo M.R. (2009). Physician Communication and Patient Adherence to Treatment: A Meta-Analysis. Med. Care.

[20] Organización Mundial de la Salud (OMS) (2007). “Nueve Soluciones para la Seguridad del Paciente” a Fin de Salvar Vidas y Evitar Daños.

[21] Soar J., Böttiger B.W., Carli P., Couper K., Deakin C.D., Djärv T., Lott C., Olasveengen T., Paal P., Pellis T. (2021). European Resuscitation Council Guidelines 2021: Adult Advanced Life Support. Resuscitation.

[22] Müller M., Jürgens J., Redaèlli M., Klingberg K., Hautz W.E., Stock S. (2018). Impact of the Communication and Patient Hand-off Tool SBAR on Patient Safety: A Systematic Review. BMJ Open.

[23] De Meester K., Verspuy M., Monsieurs K.G., Van Bogaert P. (2013). SBAR Improves Nurse-Physician Communication and Reduces Unexpected Death: A Pre and Post Intervention Study. Resuscitation.

[24] Kostoff M., Burkhardt C., Winter A., Shrader S. (2016). An Interprofessional Simulation Using the SBAR Communication Tool. Am. J. Pharm. Educ..

[25] Real Academia Española de la Lengua (RAE) RAE Diccionario de la Lengua Española. https://dle.rae.es/.

[26] Leal-Costa C., Orcajada-Muñoz I., Díaz Agea J.L., Adánez-Martínez M.G. (2022). CERCAR to COVID-19: A structured communication model for the follow-up of cases and contacts in Primary Care. Anal. Sist. Sanit. Navarra.

[27] Motola I., Devine L.A., Chung H.S., Sullivan J.E., Issenberg S.B. (2013). Simulation in Healthcare Education: A Best Evidence Practical Guide. AMEE Guide No. 82. Med. Teach..

[28] Batllori Gastón M. (2020). Simulación Clínica y La Pandemia Por COVID-19. ¿De Dónde Venimos? ¿Hacia Dónde Queremos Ir?. Anal. Sist. Sanit. Navarra.

[29] Díaz Agea J.L., Pujalte-Jesús M.J., Leal Costa C., Díaz Agea J.L., Pujalte-Jesús M.J., Leal Costa C. (2020). Simular En Tiempos de Confinamiento. Cómo Transformar La Simulación Clínica a Un Formato Online En Un Contexto Universitario de Ciencias de La Salud. Anal. Sist. Sanit. Navarra.

[30] Thomas C.M., Bertram E., Johnson D. (2009). The SBAR Communication Technique: Teaching Nursing Students Professional Communication Skills. Nurse Educ..

[31] Noh G.O., Park M.J. (2022). Effectiveness of Incorporating Situation-Background-Assessment-Recommendation (SBAR) Methods into Simulation-Based Education for Nursing Students: A Quasi-Experimental Study. Nurse Educ. Today.

[32] Creswell J.W., Clark V.L.P. (2011). Designing and Conducting Mixed Methods Research.

[33] Watts P.I., McDermott D.S., Alinier G., Charnetski M., Ludlow J., Horsley E., Meakim C., Nawathe P.A. (2021). Healthcare Simulation Standards of Best PracticeTM Simulation Design. Clin. Simul. Nurs..

[34] Del Moral I., Maestre J.M. (2013). A View on the Practical Application of Simulation in Professional Education. Trends Anaesth. Crit. Care.

[35] McDermott D.S., Ludlow J., Horsley E., Meakim C. (2021). Healthcare Simulation Standards of Best PracticeTM Prebriefing: Preparation and Briefing. Clin. Simul. Nurs..

[36] Rudolph J.W., Raemer D.B., Simon R. (2014). Establishing a Safe Container for Learning in Simulation: The Role of the Presimulation Briefing. Simul. Healthcare.

[37] Turner S., Harder N. (2018). Psychological Safe Environment: A Concept Analysis. Clin. Simul. Nurs..

[38] Cheng A., Eppich W., Grant V., Sherbino J., Zendejas B., Cook D.A. (2014). Debriefing for Technology-Enhanced Simulation: A Systematic Review and Meta-Analysis. Med. Educ..

[39] Decker S., Alinier G., Crawford S.B., Gordon R.M., Jenkins D., Wilson C. (2021). Healthcare Simulation Standards of Best PracticeTM The Debriefing Process. Clin. Simul. Nurs..

[40] Alconero-Camarero A.R., Gualdrón-Romero A., Sarabia-Cobo C.M., Martinez-Arce A. (2016). Clinical Simulation as a Learning Tool in Undergraduate Nursing: Validation of a Questionnaire. Nurse Educ. Today.

[41] Taylor S.J., Bogdan R., DeVault M. (2015). Introduction to Qualitative Research Methods: A Guidebook and Resource.

[42] World Medical Association (2013). World Medical Association Declaration of Helsinki: Ethical Principles for Medical Research Involving Human Subjects. JAMA.

[43] Díaz Agea J.L., Ramos-Morcillo A.J., Amo Setien F.J., Ruzafa-Martínez M., Hueso-Montoro C., Leal-Costa C. (2019). Perceptions about the Self-Learning Methodology in Simulated Environments in Nursing Students: A Mixed Study. Int. J. Environ. Res. Public Health.

[44] Warren J.N., Luctkar-Flude M., Godfrey C., Lukewich J. (2016). A Systematic Review of the Effectiveness of Simulation-Based Education on Satisfaction and Learning Outcomes in Nurse Practitioner Programs. Nurse Educ. Today.

[45] Mirza M.B., Sulaiman A., Hashmi S., Zaki S., Rehman R., Akbar R. (2021). Use of Simulation Based Technology in Pre-Clinical Years Improves Confidence and Satisfaction among Medical Students. JPMA J. Pak. Med. Assoc..

[46] Jiménez-Rodríguez D., del Mar Torres Navarro M., del Pino F.J.P., Arrogante O. (2020). Simulated Nursing Video Consultations: An Innovative Proposal During Covid-19 Confinement. Clin. Simul. Nurs..

[47] Jiménez-Rodríguez D., Arrogante O. (2020). Simulated Video Consultations as a Learning Tool in Undergraduate Nursing: Students’ Perceptions. Healthcare.

[48] Jiménez-Rodríguez D., Belmonte García M.T., Santillán García A., del Pino F.J.P., Ponce-Valencia A., Arrogante O. (2020). Nurse Training in Gender-Based Violence Using Simulated Nursing Video Consultations during the COVID-19 Pandemic: A Qualitative Study. Int. J. Environ. Res. Public Health.

[49] Waldner M.H., Olson J.K. (2007). Taking the Patient to the Classroom: Applying Theoretical Frameworks to Simulation in Nursing Education. Int. J. Nurs. Educ. Scholarsh..

[50] Lisko S.A., O’Dell V. (2010). Integration of Theory and Practice: Experiential Learning Theory and Nursing Education. Nurs. Educ. Perspect..

[51] Shin S., Park J.-H., Kim J.-H. (2015). Effectiveness of Patient Simulation in Nursing Education: Meta-Analysis. Nurse Educ. Today.

[52] Malhotra A., Kumar A. (2021). Breaking the COVID-19 Barriers to Health Professional Team Training With Online Simulation. Simul. Healthcare J. Soc. Simul. Healthcare.

[53] Kim J., Park J.-H., Shin S. (2016). Effectiveness of Simulation-Based Nursing Education Depending on Fidelity: A Meta-Analysis. BMC Med. Educ..

[54] Rød I., Kynø N.M., Solevåg A.L. (2021). From Simulation Room to Clinical Practice: Postgraduate Neonatal Nursing Students’ Transfer of Learning from in-Situ Resuscitation Simulation with Interprofessional Team to Clinical Practice. Nurse Educ. Pract..

[55] Hustad J., Johannesen B., Fossum M., Hovland O.J. (2019). Nursing Students’ Transfer of Learning Outcomes from Simulation-Based Training to Clinical Practice: A Focus-Group Study. BMC Nurs..

[56] Dufrene C., Young A. (2014). Successful Debriefing—Best Methods to Achieve Positive Learning Outcomes: A Literature Review. Nurse Educ. Today.

[57] Levett-Jones T., Lapkin S. (2014). A Systematic Review of the Effectiveness of Simulation Debriefing in Health Professional Education. Nurse Educ. Today.

[58] Kaplan B., Litewka S. (2008). Ethical Challenges of Telemedicine and Telehealth. Camb. Q. Healthcare Ethics.

[59] Khalil K., Das P., Kammowanee R., Saluja D., Mitra P., Das S., Gharai D., Bhatt D., Kumar N., Franzen S. (2021). Ethical Considerations of Phone-Based Interviews from Three Studies of COVID-19 Impact in Bihar, India. BMJ Glob. Health.

