# Supplementary material for: Telephone-Based Structured Communication Simulation Program for the Follow-Up of COVID-19 Cases and Contacts in Primary Care
Source: Int J Environ Res Public Health. 2022 Mar 25;19(7):3915. doi: 10.3390/ijerph19073915 (PMC8997775; doi:10.3390/ijerph19073915)
Supplement: Supplementary file 1 [file ijerph-19-03915-s001.zip › ijerph-1638387-supplementary.pdf]

Table S1.

a) Structured communication protocol *CERCAR COVID-19* for the first interview.

**C**<br>**CONTEXT**	Review the medical history.<br>We have paper and pen on hand.<br>Review the checklist.<br>We have a list of telephones of interest for the patient.<br>We ensure calmness.
**E**<br>**INTERVIEW**	Presentation, we identify ourselves.<br>We identify the patient (name, date of birth, address…).<br>We explain that the interview will last about 15 minutes.<br>We talk about confidentiality.<br>We explain that the planning we will adopt is for their benefit and that of their families and community.<br>We ask about the vaccination schedule against COVID-19.
**R**<br>**REASON FOR CALLING**	Reason for the call: we verify if the person has received the result of the COVID-19 test (if not, we provide them with the results).<br>We ask about their health, symptoms, we ask about how they are feeling.<br>We ask about the period of infection, when they started to have symptoms.<br>We explain that they must remain isolated/ in quarantine, and what this means.
**C**<br>**CONTACTS**	We explain the definition of contact.<br>We help them remember the contacts they were with 2 days before the start of the symptoms, or the PCR test.
**A**<br>**ADVICE AND ANTICIPATION**	We explain what examinations we are going to perform.<br>We explain the emergency signs they must report.<br>We provide instructions on how to isolate; we ensure that they have understood them.<br>We ensure that they can comply with the quarantine.<br>We explore worries and needs.<br>We offer resources.
**R**<br>**REVIEW**	REVIEW, QUESTIONS AND CLOSING.<br>We ensure that they have understood all the information.

b) Structured communication protocol *CERCAR COVID-19* for the follow-up interview.

**C**<br>**CONTEXT**	Review the medical history and remember if the patient is under quarantine/isolation.<br>We have paper and pen on hand.<br>Review the checklist.<br>We have a list of telephones of interest for the patient.<br>We ensure calmness.
**E**<br>**INTERVIEW**	Presentation, we identify ourselves.<br>We identify the patient (name, date of birth, address…).<br>We remind them about confidentiality.<br>We thank them for complying with the quarantine/isolation, and remind them that their effort is protecting those they love and the community in general.<br>We remind them of the vaccination schedule against COVID-19 (if needed).
**R**<br>**REASON FOR CALLING**	Reason for calling: this is a follow-up all, we tell the patients that were are aware about the implications of being isolated/in quarantine, and we value their effort.<br>We ask about their health, we ask how they are feeling.<br>We assess improvement/worsening.<br>We remind: we ask about the period of infection, and when the symptoms started.<br>We explain that they must continue with the isolation/quarantine, and ask if they have problems in doing so.
**C**<br>**CONTACTS**	We ask about their contacts, and if they have been notified.<br>We ask if they remember a contact they have not mentioned previously.
**A**<br>**ADVICE AND ANTICIPATION**	We remind them about the examinations we will conduct.<br>We remind them about the emergency signs they must report.<br>We ask them about how they are dealing with isolation; we ensure that they have understood the information.<br>We ensure that they can comply with the quarantine.<br>We explore worries and needs.<br>We ask if they need help, we remind them that they can call.
**R**<br>**REVIEW**	REVIEW, QUESTIONS AND CLOSING.<br>We ensure that they have understood all the information.
